# Experiences of male irregular migrants during their migration process and reception in Spain: lessons learned from the COVID-19 pandemic

**DOI:** 10.3389/fpubh.2024.1387715

**Published:** 2024-04-19

**Authors:** Dulcenombre de María García-López, María del Mar Jiménez-Lasserrotte, Érica Briones-Vozmediano, María Dolores Ruiz-Fernández, José Manuel Hernández-Padilla, José Granero-Molina

**Affiliations:** ^1^Faculty of Health Sciences, University of Almería, Almería, Spain; ^2^Department of Nursing, Physiotherapy and Medicine, University of Almería, Almería, Spain; ^3^Department of Nursing and Physiotherapy, University of Lleida, Lleida, Spain; ^4^Grupo de Estudios en Sociedad, Salud, Educación y Cultura (GESEC), University of Lleida, Lleida, Spain; ^5^Grupo de Investigación en Cuidados en Salud del Instituto de Investigación Biomédica de Lleida (GRECS IRB Lleida), Lleida, Spain; ^6^Facultad de Ciencias de la Salud, Universidad Autónoma de Chile, Temuco, Chile

**Keywords:** COVID-19, emergency care, government response, irregular migrant, public health, qualitative research

## Abstract

**Background:**

The causes behind migration movements are complex. The COVID-19 pandemic highlighted how several countries failed to respond to the virus adequately, while simultaneously infringing on people’s rights. Male irregular migrants fled their countries of origin and embarked on a perilous migration journey to Spain. The highly restrictive COVID-19 measures and border closures affected the mobility of male irregular migrants, whose reception in the host country posed a challenge. It led to the establishment of emergency facilities to accommodate male irregular migrants affected by COVID-19, which had repercussions on their mental health. The aim of this study was to describe and understand the experiences of male irregular migrants throughout their migration process and reception in Spain during the COVID-19 pandemic.

**Methods:**

Qualitative descriptive study. Sixteen male irregular migrants participated in this study. Data were collected between January and March 2023 through 16 one-on-one in-depth interviews. Thematic analysis was used to analyze the qualitative data using ATLAS.ti computer software.

**Results:**

Three main themes emerged: (1) How the COVID-19 pandemic drove male irregular migrants to leave their countries of origin, (2) How COVID-19 lockdown policies affected the migration journey, and (3) Receiving male irregular migrants in a pandemic: a housing labyrinth marked by isolation and loneliness.

**Conclusion:**

The COVID-19 pandemic increased the social, employment and health inequalities experienced by male irregular migrants. Border closures exacerbated the migration journey and the social stigmatization of this group, who were seen as carriers of the virus in both transit and host countries. Strict control measures in emergency and reception facilities had a significant psychological impact on the male irregular migrants due to the social isolation they experienced. Health institutions should develop programs to guarantee the care needs of irregular migrants.

## 1 Introduction

International migrant movements are characterized by a complex global interconnectedness between origin, host and transit countries (1), and exceeded 103 million people globally in 2022 (2). The causes behind migration movements are complex, and the outbreak of the SARS-CoV-2 pandemic led to an unprecedented crisis (3, 4). COVID-19 highlighted the shortcomings and incapacity of countries of origin to prepare for and respond to the virus (5). COVID-19 is a respiratory disease of varying severity; its most prevalent symptoms are a dry cough, fever, dyspnea and myalgia (6). Measures to prevent the virus included the use of masks, hand washing and social distancing (7). However, as the infection spread, most countries closed borders and limited their socio-economic activity to services considered essential (8). This magnified the difficulties faced by migrants who had no income, lived in poverty or had no access to hygiene and sanitation (9). The situation resulted in health, economic and social collapse, which led migrants to leave their countries as they were unable to cope with this systematic vulnerability (10). As a result, the number of people who migrated irregularly during the pandemic increased by 154% in Central Mediterranean countries and by 46% in Spain (11) compared to previous years. One of the most common routes for migrants entering Europe is by sea through Spain’s southern border (12). Many of these people are irregular migrants (13) who come from African and sub-Saharan African countries (14). The term irregular migrant (IM) refers to a migrant who has no legal permit, documentation, or refugee status, and is not authorized to enter or stay in a given country (15). In 2023, 50,551 IMs arrived in Spain (16), undertaking a dangerous journey in small boats (17). The IMs’ experiences during their migration journey had a negative impact on their health (13). IMs lacked sufficient resources to be able to mitigate the consequences of the pandemic (18, 19). Their legal status was an obstacle to accessing regularized employment or state benefits (20), thus increasing their exposure to the virus (21, 22). They also had poor living conditions (23), which made it impossible to comply with virus prevention measures (24). IMs were perceived in some cases as a threat to the local population (25). This discrimination had an impact on their mental health, increasing levels of stress (26), anxiety and depression (27, 28). This was compounded by loneliness and the difficulty of accessing the country due to border closures during the COVID-19 pandemic (29).

The COVID-19 pandemic posed a challenge for countries receiving large numbers of migrants, as the structural inequalities faced by the most vulnerable groups were exacerbated (5, 30). Upon arrival in Spanish territory, the IMs were intercepted by the State Security Forces and cared for by Non-Governmental Organizations (NGOs) who were in charge of initial emergency care (12). Healthcare providers had to change the way they worked because of COVID-19 (31). The Detention Centers for Foreigners (DCF) were closed and transfer to Humanitarian Reception Centers (HRC) (32) immediately after police custody was not possible. Therefore, Shared Management Facilities (SMFs) were established as accommodation to confine IMs who had an active COVID-19 infection or had been close contacts (25). These temporary and sometimes makeshift emergency facilities largely overlooked the needs of IMs (33). While there are studies on the impact of COVID-19 on the health system and its impact on IMs (15, 34), as well as IMs’ access to resources such as vaccines (35**–**37), few studies have analyzed the experiences of male irregular migrants (MIMs) during the migration journey and reception in the host country during the pandemic. The aim of this study was to describe and understand the experiences of male irregular migrants throughout their migration process and reception in Spain during the COVID-19 pandemic.

## 2 Methods

### 2.1 Design

A descriptive qualitative study was carried out. This type of design allows the study phenomenon to be described in a way that captures the participants’ narratives as closely as possible (38). This approach is based on naturalistic research principles and allows for the exploration of hitherto underexplored phenomena within a specific context (39). For this reason, a study with a descriptive qualitative design was suitable for understanding the experiences of MIMs who reached Spanish shores by boat during the COVID-19 pandemic (40). The Consolidated Criteria for Reporting Qualitative Research were followed (COREQ) (41).

### 2.2 Participants and context

This study took place in an HRC located in a province in southern Spain. The participants were recruited using convenience sampling. Inclusion criteria were: (1) to be a male irregular migrant, (2) to have reached the Spanish coastline by boat during the COVID-19 pandemic, (3) to be African, (4) to be 18 years of age or older, and (5) to have stayed in an HRC. The exclusion criteria were: (1) to have cognitive impairment and (2) to demonstrate a complete lack of understanding of the research topic. For the recruitment of the sample, we were assisted by a team of psychologists from an NGO, who acted as a bridge between the researchers and the participants interested in the study. The researchers personally contacted the participants interested in the study. They explained the objectives of the research and asked for voluntary consent to participate in the study. Twenty-one MIMs who had stayed in the HRC during the pandemic were invited to participate, five of whom declined due to a lack of time. The final sample consisted of 16 male irregular migrants. The mean age was 24.2 years and the minimum length of stay in the HRC was 2 months (Table 1).

**Table 1:** Sociodemographic characteristics of the participants (*N* = 16).

Participant	Age	Sex	Country of origin	Religion	Months in HRC	Ethnicity
IDI1	21	M	Morocco	Muslim	14	Amazigh
IDI2	26	M	Senegal	Muslim	2	Wolof
IDI3	20	M	Mali	Muslim	24	Soninke
IDI4	28	M	Morocco	Muslim	7	Amazigh
IDI5	26	M	Senegal	Muslim	12	Serer
IDI6	37	M	Mali	Muslim	12	Soninke
IDI7	20	M	Guinea	Muslim	5	Soninke y Susu
IDI8	22	M	Morocco	Muslim	8	Amazigh
IDI9	24	M	Mali	Muslim	6	Soninke
IDI10	26	M	Senegal	Muslim	4	Wolof
IDI11	22	M	Morocco	Muslim	12	Amazigh
IDI12	23	M	Morocco	Muslim	7	Amazigh
IDI13	25	M	Nigeria	Muslim	3	Yoruba
IDI14	20	M	Ivory Coast	Muslim	4	Baoule
IDI15	25	M	Cameroon	Muslim	4	Tikar
IDI16	22	M	Algeria	Muslim	7	Amazigh

IDI, In-depth Interview; M, Male; HRC, Humanitarian Reception Center.

### 2.3 Data collection

The data collection included 16 one-on-one in-depth interviews (IDIs) conducted between January and March 2023. The IDIs took place in an office of the HRC where the MIMs lived for a few months after their arrival in Spain. The IDIs were carried out by several researchers trained in qualitative research, following an interview protocol (Table 2). Before starting the IDIs, the participants’ socio-demographic data were collected and informed consents were signed. Each participant participated in only one IDI that lasted approximately 60 min. They used both Spanish and Arabic with the help of cultural mediators. The IDIs were audio-recorded for later transcription into Spanish and analysis by the research team. Data collection ended when no new information was provided and data saturation had been reached.

**Table 2:** Interview protocol.

Phase	Theme	Content/example questions
Presentation	Purpose	To understand male irregular migrants' experiences of their migration journey during the COVID-19 pandemic.
	Ethical considerations	Inform about the voluntary nature of participation, consent, the possibility of withdrawal and confidentiality.
Opening	Opening question	“How has the COVID-19 pandemic affected your country of origin?”
Development	Specific questions	“What were the consequences of the border closure and how did it affect your migration journey?” “What COVID-19 protocols were followed by the people who rescued when you arrived in Spain?” “How were your basic needs met during lockdown?”
Closing	Final question	“How could the health care you received be improved?”
	Acknowledgements	“Thank you for your time. Your participation will be very useful.”
	Future contact	“We would like to remind you that you can contact us with any questions you may have. When we have the results of the study, we will inform you.”

### 2.4 Data analysis

Data analysis was carried out using ATLAS.ti.23. The data were analyzed following the thematic analysis described by Braun & Clarke (42): (1) Familiarization with the data: a complete reading of all transcripts was carried out to extract the general meaning of the participants’ narratives, followed by a re-reading to write annotations using the “add memo” function in ATLAS.ti. (2) Systematic coding of the data: significant quotes were selected together with their respective code assignment using the “open coding” and “*in vivo* coding” function in ATLAS.ti. (3) Generation of initial themes from the codes and data collected: codes with shared meaning and linked by a key idea were grouped together to generate representative themes (Table 3). (4) Development and revision of themes: researchers checked that the established themes were consistent with the grouped codes and the quotes coded by them. (5) Refining, defining and naming themes: themes were reviewed again to refine the analysis, and the wording of the final themes was established. (6) Report writing: the researchers selected the most relevant quotes and carried out synthesized descriptions of each theme and sub-theme. Key aspects of the analysis were related to the research question and available literature on the subject.

**Table 3:** Themes, subthemes and condensed meaning units.

Theme	Sub-themes	Units of meaning
How the COVID-19 pandemic drove male irregular migrants to leave their countries of origin	Factors for migration: “Escaping the COVID-19 crisis”	Economic consequences, employment consequences, confinement, movement restrictions, overcrowding.
	The MIMs’ perception of and capacity to deal with COVID-19	Fear of disease, denial, pandemic informants, hoaxes, mask, resources, experience of disease.
How restrictive COVID-19 policies affected the migration journey	Increased control measures and stigmatization during the pandemic	Insufficient food and water, lack of petrol, extreme weather, neglected health, racism.
	Making the crossing by boat was no easy feat	Lack of resources, small boat, safety distance, death.
	Emergency care for newly arrived MIMs: COVID-19 health alert	Cultural mediators, nurse, COVID test, police custody, COVID protocol.
Receiving MIMs in a pandemic: a housing labyrinth characterized by loneliness and isolation	Shared management facilities: hotels and emergency shelters for MIMs affected by COVID-19	Housing, COVID positives, close contact, needs, social exclusion, fear, racism.
	The pandemic affected the social integration of MIMs in shelters and child protection centers	Humanitarian reception center, integration, appreciation, isolation, minors.
	The emotional and health repercussions of COVID-19: the pandemic paralyzed my migration journey	Mental health, fear, vital distress, disbelief, family support, absence.

### 2.5 Rigor

Strategies based on Lincoln and Guba’s criteria were used (43). Credibility: the team of principal investigators was composed of reputable professionals with a long track record in qualitative research and immigration. Dependability: The strategy of data triangulation was used through data verification by several researchers. In addition, a detailed description of the study’s objective and methodology was provided. Transferability: the participants’ narratives were described in detail until data saturation was reached. Confirmability: the transcripts and a table summarizing the results were given to the participants to confirm their accuracy and interpretation.

### 2.6 Ethical considerations

This research was conducted in accordance with the ethical principles of the Declaration of Helsinki (44). Permission was obtained from the Research Ethics Committee of the University of Almeria (Anonymized). All participants were informed of the objective, methodology, voluntary nature of participation and possibility to withdraw from the study at any time. Signed informed consent was obtained from all participants. Confidentiality and anonymity were guaranteed in accordance with Organic Law 3/2018, of 5th December, on Personal Data Protection and Guarantee of Digital.

## 3 Results

Three main themes and eight sub-themes were drawn from the data analysis (Table 3). These themes and sub-themes provided insight into the experiences of MIMs who arrived on the Spanish coast by boat during the COVID-19 pandemic.

### 3.1 How the COVID-19 pandemic drove male irregular migrants to leave their countries of origin

This theme focuses on describing the main reasons why MIMs left their respective countries of origin during the pandemic, ranging from financial factors to health or social issues. The participants also described how they were informed about COVID-19 as well as their perception of the disease when the first positive cases were reported.

#### 3.1.1 Factors for migration: “escaping the COVID-19 crisis”

The MIMs experienced various traumatic experiences in their countries of origin that drove them to migrate, such as a persistent attack on their rights. Some of them explained how losing their parents left them in a situation of extreme social vulnerability. They suffered from mistreatment and persecution on a personal level, which triggered their departure. Moreover, social problems related to war were another cause, and in some cases, even slavery was made evident.

“*There was a social problem, because in the area where we lived (Mali) there was an inter-ethnic conflict. There are some people who consider others as slaves. We want to fight against that, but as we are a minority, we don't have the power to stop this phenomenon.”* (IDI9-Mali).

The COVID-19 pandemic led to the reorganization of the health system worldwide, which had an impact on the public. The participants were unanimous in explaining that resources to fight the virus were limited or non-existent in their respective countries of origin. Many of them described reduced access to health care due to overcrowded hospitals and the collapse of emergency departments. In addition, MIMs lacked sufficient purchasing power to access private healthcare services. As a result, they felt they were fully exposed to the virus.

“*There was a lot of COVID in my country (IM), many people died and the hospitals collapsed. Not everyone has access to healthcare, you have to have money in your pocket. Not everyone had access to a mask either. I wanted to get out of there”* (IDI5-Senegal).

According to the participants, fear of contagion, along with confinement and other movement restrictions, had an impact on employment. Some companies reduced or withheld wages from workers, resulting in emotional stress as financial uncertainty loomed. The MIMs explained that they did not receive any income for a long period of time and had to rely on acquaintances to survive. As a result of the confinement, many businesses closed, and the public began to get into debt. Governments offered monetary aid in an attempt to deal with the damage caused by the pandemic, but it was insufficient.

“*I was working in a private school. When COVID started we couldn't work and we had a lot of financial problems. I had to wait four months to get paid and they only paid me 50%”* (IDI4-Morocco).

#### 3.1.2 The MIMs’ perception of and capacity to deal with COVID-19

The MIMs cited the police or the media, such as radio or television, as the main informants at the beginning of the pandemic. Keeping a safe distance, wearing gloves, and using masks were preventive measures disseminated by the media. They also reported on the number of infected people and deaths from the virus. The participants highlighted the media’s important role in raising awareness about the pandemic.

*“One day I went out with my friends and the police came to tell us that the next day we all had to stay at home because there was COVID. There was a total lockdown for everyone. There was no going to work or anything. We were caught by surprise”* (IDI7-Guinea).

When the participants were asked about the protective measures in place in their countries, the vast majority described the mandatory use of face masks in public spaces. The MIMs in the study also felt it was important to respect timetables and curfews. Vaccines became available to the whole population, but some MIMs refused to be vaccinated. One of the participants stated the importance of respecting the most vulnerable groups in the population:

"*I had a lot of information about COVID. I know that it spreads quickly among people. You have to be careful with the older and children as they are more likely to be the worst affected”* (IDI4-Morocco).

The participants perceived that fear was prevalent in society, especially in the midst of uncertainty about the resources available to fight the disease. The MIMs sometimes held negationist stances due to the media’s lack of credibility. However, this perspective would change in the event of a direct experience with the virus. In the meantime, the MIMs’ loved ones actively raised awareness, especially if there was a sick person in the family or if they were in different places.

*“Before, I used to say: ‘It doesn't make sense to me,’ because they always said there were dead people and I wasn't seeing any dead people. But the day I caught COVID, very dangerous. It can kill a lot of people. I didn't sleep that night, I had a high fever, pain in my joints, a lot of pain. My family suffered terribly, not knowing if there was medicine or a solution. You had to be scared!”* (IDI2-Senegal).

### 3.2 How restrictive COVID-19 policies affected the migration journey

This theme reveals how COVID-19 had an impact on the participants’ migration journeys. The increased control measures during the pandemic forced the MIMs to change their route, thus making them more vulnerable. In addition, the MIMs’ key priority during the boat journey was survival, which overshadowed any concerns they may have had about being affected by the virus.

#### 3.2.1 Increased control measures and stigmatization during the pandemic

Reaching the North African countries was a challenging endeavor. Some MIMs had to cross deserts in extreme temperatures with hardly any food or shelter. The pandemic meant that this arduous journey was prolonged by border closures between countries. In some cases, the IMs were forced to rearrange their migration route or even start the journey again in the event of being deported. In general, their time in North African countries was prolonged, thus their suffering was too.

“*It took me six months to get from Senegal to Spain. COVID meant that we had to change routes because the borders were closed. You had to pay at every border with the risk that if you were caught you would be sent back. In Tangier we were scared and had to transfer to Nador”* (IDI5-Senegal).

The COVID-19 containment measures established in transit countries had an impact on the MIMs. Compulsory confinement led the participants to feel hopeless and lonely, as they had no way of contacting other compatriots or making a living to survive. Most of the MIMs were unable to work due to the informal nature of their jobs. As a result, they resorted to charity work or scavenging food from rubbish bins to make ends meet.

“*During COVID I was in Morocco. Tt was very difficult for me because there was a lot of control. We couldn't sell anything or work. There were people who gave me a hand to help me to live”* (IDI2-Senegal).

Some participants felt completely rejected by the local population during their time in transit countries. The spread of the pandemic gave rise to prejudice and increased discriminatory attitudes towards the participants. Some MIMs experienced racist behavior and were seen as COVID-19 disease spreaders during their migration journey, which made them feel frustrated. In addition, the absence of a stable support network throughout the journey increased the MIMs’ feelings of loneliness and helplessness, which had a negative impact on their mental health.

"*People said that because there were a lot of black people, all kinds of diseases were coming in. I said it wasn't our fault, it wasn't just happening in Algeria, it was happening all over the world. To say that black people were bringing COVID or other diseases is nonsense, it's frustrating”* (IDI7-Guinea).

#### 3.2.2 Making the crossing by boat was no easy feat

The participants explained that once they had managed to cross the various borders, they were faced with the new challenge of having to cross the Mediterranean Sea in a small boat. Before embarking on the journey, they were aware that it was going to be dangerous and risky. Furthermore, there was a severe lack of resources, which made it even more unpleasant for the MIMs. Food and water ran out along with petrol, leaving them adrift for several days, sometimes resulting in death.

“*It was long and it was very hard, because travelling by boat at sea is not easy. There is a lot of risk. We also had to go without food and nothing for a long time, and one boy died because there was no water left, poor thing"* (IDI6-Mali).

On many occasions, the MIMs had to contact mafias who took advantage of their plight. The participants described how they were required to pay large amounts of money, which were disproportionate to the service offered. Excessive numbers of MIMs were forced to travel on the same boat, increasing the risk of capsizing. The participants felt scared and unsafe in this situation. The overcrowding of many people on a single boat also posed a risk to their health as they were unable to keep a safe distance between one another. However, the risk of becoming infected during the journey was not a priority; what they really wanted was to reach Spain alive.

“*I paid 1500 euros to the mafia, got on a boat with 18 strangers. It took us seven days to get there, on the third day we ran out of food and water. I didn't care about COVID, none of us wore masks and when we arrived, three guys were positive for COVID”* (IDI4-Morocco).

#### 3.2.3 Emergency care for newly arrived MIMs: COVID-19 health alert

After several days of crossing, some of the MIMs were rescued by maritime rescue teams. This was a joyous moment for the participants as they felt safe and saw their dream of reaching Spain come true. After disembarking at the nearest port, they were attended to by Red Cross teams. Cultural mediators and nurses provided the new arrivals with health and humanitarian care to help them recover from the grueling journey. The COVID-19 health protocol meant that the care process included high levels of safety, protection and testing for the disease.

*“The boat ran out of petrol, we called an NGO from Morocco and it was Maritime Rescue who saved us. I was scared, but I felt safe because we had been rescued! They took us to some facilities, where we were attended to in a protective suit. First, they gave us the COVID-19 test, we showered, they gave us food and clean clothes - you had to wear a mask, it was compulsory! I felt reassured by the nurses because they spoke French and gave us information about the COVID-19 tests”* (IDI6-Mali).

After initial health checks, the MIMs remained in police custody, ranging from a minimum of a couple of hours to a maximum of three days. The police focused on the identification of the participants along with the temporary removal of their possessions. The participants indicated that the only mandatory pandemic measure during their time in the cells was to wear a mask. The MIMs perceived that their needs were being attended to, but it was nonetheless an unpleasant experience, marked by sadness at not being able to contact their families and tell them that they had arrived in Spain. In some cases, the MIMs’ families were not even informed that they were going to undertake the journey.

*“Yes, it had everything. I slept well because they give us a big mattress, but it was a really hard few days. I couldn't get in touch with my family because they had taken everything away from us. I left Morocco, no one in my family knew I was going to leave. I was worried about my family”* (IDI4-Morocco).

After remaining in police custody, MIMs are usually referred to shelters or immigration detention centers. However, the COVID-19 pandemic restricted the participants’ access to such facilities, thereby limiting their quality of life. Statements from some of the participants expressed feelings of fear and deep sadness at being forced to live on the streets for an indefinite period of time in an unfamiliar country.

“*The police gave us a piece of paper and told us everyone to get out on the street, that the Ministry has no places to sleep and we can't enter any center. I told him that I didn't know where to go. We were in the street, in the cold. I was happy because they had given me my freedom and I was here, but I had no idea of the suffering that awaited me”* (IDI7-Guinea).

### 3.3 Receiving MIMs in a pandemic: a housing labyrinth characterized by loneliness and isolation

This issue describes the reception process for when MIMs arrived in Spain during the pandemic. After the first few hours of precautionary detention in police custody, they were released and supposed to transferred to Immigration Detention Centers (IDCs) or Humanitarian Reception Centers (HRCs). However, in the COVID-19 pandemic scenario, the closure of IDCs and the impossibility of direct referral to HRCs, meant that they were referred to Shared Management Facilities (SMFs) instead, where the established quarantines could be carried out. The MIMs expressed feelings of loneliness and fear during their stay in the different accommodation facilities, which had repercussions on their health and emotional wellbeing.

#### 3.3.1 Shared management facilities: hotels and emergency shelters for MIMs affected by COVID-19

Due to precarious migration conditions, many of the participants tested positive for COVID-19 or were close contacts of a positive person upon arrival in Spain. During the COVID-19 pandemic, the health authorities were responsible for complying with health care regulations by providing housing resources to the MIMs who had recently arrived in Spain by boat. This made it impossible to deport them to their countries of origin or to place them in humanitarian reception centers for migrants. The public health system and NGOs worked together to set up SMFs to cover the accommodation and health needs of IMs affected by COVID-19. The participants were referred to different hotels and shelters throughout the country for the confinement period. Their experiences of the services, facilities and security measures varied greatly. This is how several of the participants described it:

*“In Las Palmas, Gran Canaria, I was in a kind of sports center. About 60 of us slept in a big room, there was no safety distance, we slept next to each other. There were a lot of COVID problems. They say that 30 had COVID, they isolated them and the rest of us had to quarantine. But then they take them out of isolation and they keep going back in and out. Terrible!”* (IDI8-Guinea).

*“We were taken to a hotel, four people per room. We always had to wear a mask, wash our hands with gel and our temperature was taken at the restaurant door. The rules of the center were explained to us in French when we arrived at the hotel and a nurse came to treat our wounds. I did two quarantines. The first quarantine was for 15 days and another quarantine because we were told that there was a positive case in the hotel. There were more than 500 people in the hotel. They were good, I was able to call my father!”* (IDI9-Mali).

During their stay in these shelters, the participants faced multiple challenges, including social exclusion, marginalization and fear. Some MIMs highlighted that because of their undocumented migrant status or nationality they were subjected to racism and intimidation. These situations were intensified by social isolation, the impossibility to carry out leisure activities or to obtain financial support for their basic needs.

*“We stayed in a hotel in Maspalomas for almost a month in August 2020. We were allowed to go out from six in the morning until ten at night. Before we got there, we were tested, and we were all negative. When we arrived, they put us in isolation for a week because the people in the village didn't know any foreigners. A lot of us were black, Moroccans and Algerians. People said that there weren't many black people there”* (IDI8-Guinea).

*“You spend many weeks in isolation, doing nothing, and you feel miserable. They only gave us food, but we had nothing to entertain ourselves. I smoke and I had to go out and pick up cigarette butts so I could smoke. They didn't give us any money. When we asked for something, they would send the police to intimidate us. They would come and say: "You don't know how many people here are suffering and you eat and pay nothing, you sleep and pay nothing”* (IDI3-Mali).

#### 3.3.2 The pandemic affected the social integration of MIMs in shelters and child protection centers

After completing their quarantines in the SMFs, some MIMs were granted a place in a humanitarian reception center for a maximum stay of three months. These facilities aimed to meet the MIMs’ basic needs, provide them with accommodation, and promote their social and professional integration. The participants expressed feelings of gratitude for the treatment they received and for all the COVID control measures in place. They felt protected and that all their needs were met. However, the confinement situation negatively affected their mental health status; they felt lonely as a result of social isolation.

*“I was in a Red Cross center. It was perfect, we had everything, people cared about us. They took a lot of precautions due to COVID. I had to do another ten-day quarantine, that's when I got COVID. I was sick with a cough, a cold and a headache. They showed me how to go to the health center and they issued me a health card. It was hard because I couldn't go out, I was always in the center, never able to go out, never able to talk to my friends, it was difficult”* (IDI7-Guinea).

One of the participants was a minor when he arrived in Spain. In these cases, the Public Prosecutor’s Office for minors is informed, and he was taken into the Child Protection System. During his stay in a child protection center, a state of alarm was declared, and he was confined for months. The participant expressed feelings of loneliness and neglect that stemmed from prolonged social isolation. He also felt scared due to a lack of information surrounding his health and because nobody explained the medical tests he was undergoing in a way that he could understand. He described it in the following way:

“*I got here and that same night I slept in a child protection center. There was no COVID protocol because I arrived in February 2020. That was until lockdown! I felt very awful, you have just arrived in a new country and you feel like a prisoner. Many of my peers told me not to accept having my blood taken, they took a lot. They sent you for pee and poo tests, it was too much, they should tell us why they are testing us, I was very scared!”* (IDI8-Guinea).

#### 3.3.3 The emotional and health repercussions of COVID-19: the pandemic paralyzed my migration journey

From the moment they arrived in Spain, the participants moved through different accommodation facilities including SMFs, HRCs and child protection centers. During this period, they were subjected to multiple diagnostic tests and quarantines in order to ensure the effectiveness of COVID containment measures and to prevent possible imported cases. These protocols in the accommodation facilities extended both the length and number of quarantines, which affected the MIMs’ mental health. The participants voiced feelings of loneliness, fear and emotional distress due to prolonged social isolation. One participant highlighted the psychological help received at an HRC:

*“I went through several accommodation facilities, Hierro, Tenerife, Malaga and Almeria. In the first one in El Hierro I spent 25 days in isolation in a room alone, unable to go out. I know they did it to protect us, it was the protocol, but I was scared and depressed. In one of the centers, they assigned me a psychologist and little by little I calmed down”* (IDI9- Mali).

The COVID-19 pandemic made the participants feel scared and concerned. While they were initially skeptical about the negative implications of the disease, when they fell ill, they became aware of the health and social repercussions. Some of them spoke about how scared they were of this unknown disease as well as their lack of support from family or peers to deal with the situation.

*“I have been isolated more than four times and I have had a lot of tests done up my nose, I thought it was going to burst. I had a pain in my chest and I thought it was tuberculosis. I went to the hospital, they tested me for COVID, AIDS and TB. The doctor told me everything was fine, negative. Then I went to the hotel and they told me I had to isolate myself. I had a friend, but when I told him I was in isolation with chest pains, he stopped talking to me. It was the worst!”* (IDI8-Guinea).

COVID-19 affected all spheres of life. Most of the participants agreed that their main concern was their family. They came to Spain to make a living and work, so that they could financially support their families in their country of origin. However, the pandemic disrupted their migration plans. Their sadness was palpable as they recalled the difficult experience of arriving in a new country, and their inability to find a job or to communicate with their loved ones.

*“I am no longer afraid of COVID because I have been vaccinated, but I had a hard time because of my family. It was ten days before I was able to send my father a message to let him know that I was well. I was in isolation and didn’t have a phone. Thanks to a nurse who wanted to help me, I was able to send a message from her mobile phone. By the time I was able to buy a phone, I had 390 messages from my family!”* (IDI4-Morocco).

*“I was not afraid of COVID, but the pandemic made things difficult. I am a father, and I came to Spain to look for work. The confinement and not being able to go out made it difficult to find a job. It was very hard!”* (IDI6-Mali).

## 4 Discussion

The aim of this study was to describe and understand the experiences of male irregular migrants throughout their migration process and reception in Spain during the COVID-19 pandemic. This qualitative descriptive study has allowed us to understand the experiences of MIMs in their migration process up to their arrival in Spain. The COVID-19 pandemic increased the vulnerability of MIMs, forcing them to leave their countries of origin. The participants undertook a dangerous migration journey, crossing borders with strict control measures until they reached Spain by boat. In the host country, the participants lived in different accommodation facilities where they underwent multiple diagnostic tests and preventive quarantines in order to adhere to containment measures and prevent possible imported cases of COVID-19. The increase in migration movements to Europe highlights the importance of identifying the needs of MIMs, as well as how to meet them (20). MIMs initiated the migration process due to financial factors (45), family troubles, lack of public safety (46) or armed conflict (47). Migrant women, in particular, fled a culture of exploitation and gender-based violence in their countries of origin (48). COVID-19 worsened the pre-existing conditions of the most vulnerable population groups, and exacerbated inequality and marginalization (21). Consistent with other studies, MIMs faced specific challenges brought about by the COVID-19 pandemic (49). The MIMs’ exposure to the virus and infection rates were higher. The pandemic increased health, economic and employment inequalities, (50, 51) which limited access to resources required to protect oneself from COVID-19 (25). This study has highlighted the fragile health systems in the countries of origin, poor health coverage and the shortage of beds for COVID-19 patients (52, 53). The results have shown that many MIMs were working in informal sectors incompatible with the remote work encouraged by the confinement measures (21). The imposition of movement restrictions meant that the MIMs lost jobs and therefore income (54). They were unable to provide for their families, which had devastating consequences on their wellbeing (55). The results of this study suggest that the participants’ perceptions of the risk associated with SARS-CoV-2 infection were diverse and changed as the pandemic evolved. Similarly, other studies highlight how the initial perception of COVID-19 posing a low risk was influenced by the information received in their countries of origin (56). Limited awareness of the virus, as well as mistrust of the government and authorities, had an impact on the effectiveness of control measures (57). MIMs who had a close experience with COVID-19 became more negative about the possibility of contracting the disease, thus changing their perception of risk (58).

As the results have shown, IMs wishing to reach Europe made the migration journey via irregular routes (59), and were subject to adverse weather conditions, dehydration and malnutrition (13). Irregular migration involves crossing borders without documentation or residing in transit areas without visas or employment agreements (60). To prevent COVID-19 transmission, measures such as border closures (61) were implemented, which prolonged the MIMs’ time in transit countries. Consistent with other studies conducted in African countries and along the border connecting Central America with the United States, most MIMs worked in the informal economy and were therefore unable to find work (53). As a result, they were exposed to violence, discrimination and racism (62). Studies suggest an increase in stigmatization and xenophobia worldwide since the onset of the COVID-19 pandemic (63, 64). In line with the findings of this study, others have indicated that MIMs are at an additional risk of being blamed for the spread of the SARS-CoV-2 virus (30). This study has shown how participants embarked on a dangerous journey by boat to reach the Spanish coast. The MIMs put their integrity at risk, travelled in overcrowded conditions without safety measures and suffered from dehydration, hunger, burns, injuries and anxiety (65). The MIMs were rescued by maritime rescue services, who, according to the SAR Convention, are obliged to take the migrants to the nearest safe port (66). Upon arrival, they were cared for by NGOs who provided the health and humanitarian care required to meet their most basic needs (65, 67). These teams were staffed by nurses who carry out clinical, humanitarian and social assessments (16). The emergency care teams implemented new measures and protocols to improve clinical care, such as diagnostic methods to detect IMs affected by COVID-19 and isolation of their close contacts (68). In line with other studies, the participants were positive about their experience of health care and cultural mediation as it allowed them to have a better understanding of the health protocols and control measures established for this new disease (65). After receiving initial emergency care from NGOs at the Spanish border, MIMs had to remain in police custody for a maximum of 72 h for profiling and deportation procedures (69). The protocol established by the National Police was to house all MIMs arriving on the same boat in the same cell. Due to the limited capacity in terms of number and size of the cells to hold the detainees, it was difficult to implement preventive and self-protection measures such as social distancing, thus increasing the risk of contracting COVID-19 (70).

The Humanitarian Reception System was set up to manage the reception of IMs arriving on Spanish shores. After their release and in light of their vulnerable situation caused by a lack of social, family and financial support, the MIMs were referred to reception facilities (33). IMs with COVID-19 and their close contacts were guaranteed care during the pandemic thanks to the establishment of Shared Management Facilities, where the IMs were quarantined under the responsibility of the Regional Health Ministries (26). Consistent with other studies, the participants highlighted the negative experience of their time in these facilities due to the poor living conditions in some of the hostels and halls where they stayed. This included overcrowding and a climate of mistrust, discrimination and reprisals (70). The imposed measure of solitary confinement had a significant psychological impact on the MIMs (71). They experienced psychological distress, fear of contracting the disease, uncertainty about the future, involuntary confinement or concern for their loved ones (72), all while having to come to terms with their failed migration plans (30). Despite these challenges, the participants felt safe and were grateful for the care and psychological support they received in the reception facilities, especially the HRCs (73).

### 4.1 Limitations

This study has several limitations that should be considered when interpreting the results. All participants were men from different African countries; other nationalities or genders might yield different results. The researchers were accompanied by cultural mediators during the interviews who spoke the languages most commonly used by the participants. However, the variation in dialects and cultural norms could influence the understanding of the participants’ experiences. All interviews were conducted at the HRC, as it was considered a familiar and easily accessible place for the participants, but this may contribute to the limitations of the study.

## 5 Conclusion

The COVID-19 pandemic had an impact on MIMs’ finances, employment and health in their countries of origin. The inability to work, the resulting lack of income and the collapse of health services increased pre-existing inequalities. During their migration journey, the MIMs had very few resources and were exposed to extreme weather conditions and violence. Due to border closures, the MIMs were stuck in transit countries with strict control measures. In addition, they were targeted as being virus carriers, leading to increased social stigmatization. Upon their arrival in Spain, the MIMs were rescued and cared for by emergency teams, who established clinical protocols to test for the COVID-19 virus. The MIMs highlighted the clinical and humanitarian role of the nurses who, together with the cultural mediators, helped them to understand the control measures for this new disease. In order to comply with healthcare regulations, the MIMs affected by COVID-19 were referred to SMFs, which had a significant psychological impact on them as social isolation led them to feel lonely. After completing quarantine, the MIMs were referred to HRCs where both their basic and housing needs were met. The MIMs discussed the biopsychosocial care received by the HRC professionals, which helped to reduce their emotional distress and make them feel safer and more in control against COVID-19. Training for healthcare professionals based on equity and social justice is recommended to improve the care provided to IMs during the different phases of the migration journey. Understanding the experiences of MIMs who arrived on Spanish shores during the COVID-19 pandemic could help health systems to develop health programs that guarantee better quality care for this group of people.

## Data availability statement

The original contributions presented in the study are included in the article/supplementary material, further inquiries can be directed to the corresponding author.

## Ethics statement

The studies involving humans were approved by Research Ethics Committee of the University of Almeria. The studies were conducted in accordance with the local legislation and institutional requirements. The participants provided their written informed consent to participate in this study. Written informed consent was obtained from the individual(s) for the publication of any potentially identifiable images or data included in this article.

## Author contributions

DG-L: Conceptualization, Investigation, Methodology, Project administration, Resources, Validation, Visualization, Writing – original draft, Writing – review & editing. MJ-L: Conceptualization, Funding acquisition, Investigation, Methodology, Project administration, Resources, Validation, Visualization, Writing – original draft, Writing – review & editing. ÉB-V: Formal analysis, Methodology, Writing – original draft. MR-F: Data curation, Software, Supervision, Writing – review & editing. JH-P: Data curation, Formal analysis, Supervision, Validation, Writing – review & editing. JG-M: Conceptualization, Methodology, Project administration, Resources, Software, Supervision, Validation, Writing – original draft, Writing – review & editing.
